# Pathological characteristics of liver injury induced by *N*,*N*-dimethylformamide: From humans to animal models

**DOI:** 10.1515/med-2022-0609

**Published:** 2022-12-09

**Authors:** Haicong Wu, Yixian Shi, Yongqin Yan, Jiaxiang Zhang, Xiaoling Zhou, Xuan Mei, Zhiyong Zheng, Dongliang Li

**Affiliations:** Department of Hepatobiliary Medicine, 900th Hospital of Joint Logistics Support Force, Fuzhou, Fujian, China; The Second School of Clinical Medicine, Southern Medical University, Guangzhou, China; Department of Respiratory, Mengchao Hepatobiliary Hospital of Fujian Medical University, Fuzhou, Fujian, China; Department of Pathology, 900th Hospital of Joint Logistics Support Force, Fuzhou, Fujian, China; Department of Medical Oncology, Zhangzhou Affiliated Hospital of Fujian Medical University, Zhangzhou, Fujian, China

**Keywords:** *N*,*N*-dimethylformamide, liver failure, animal model, clinical pathology, heterogeneous lesions

## Abstract

*N*,*N*-Dimethylformamide (DMF) is widely used in chemical industries because of its excellent solvent properties. Poisoning accidents caused by DMF have been frequently reported, particularly hepatotoxicity; however, the hepatic pathological changes have rarely been described. This study aimed to summarise the pathological characteristics of the hepatotoxicity associated with DMF in clinical cases and to verify in animal models. Liver pathologies of two patients with liver failure due to DMF were retrospectively analysed. Thirty-six rats were categorised into the DMF group (intraperitoneally injected with 4 g/kg DMF once a week), carbon tetrachloride (CCl_4_) group (intraperitoneally injected with 0.5 g/kg CCl_4_ twice a week) and control group (intraperitoneally injected with normal saline once a week). The general condition and changes in hepatic pathology at 48 h and 8 weeks were observed. Liver tissues of patients exhibited multiple unevenly distributed inflammatory and fibrotic lesions. The DMF-induced liver injury animal model was successfully established. Inflammation and fibrosis were heterogeneously observed throughout the liver in the DMF group, contrast to entirely homogeneous lesions in the CCl_4_ group. Specific hepatic pathological findings (heterogeneous lesions) caused by DMF detected for the first time in humans and animal model, may be significant in the clinical diagnosis of DMF poisoning.

## 1 Introduction

*N*,*N*-Dimethylformamide (DMF) is a broad-spectrum chemical compound and an excellent solvent, primarily used in industries involved in organic synthesis, inorganic chemical industry, and the production of synthetic fibre and artificial leather. Because DMF is a colourless, amine-flavoured, and volatile liquid, it can be absorbed into the body through the skin and respiratory tract in the workplace [1]. Toxic effects of DMF were reported [2,3,4,5] to harm the liver, gastrointestinal tract, kidney, respiratory system, reproductive system, immune system, and nervous system. The injury mechanism involves lipid peroxidation [6], mitochondrial damage [7], calcium homeostasis disorder [8], as well as cell apoptosis [9,10], proliferation, and differentiation [11]. A cross-sectional study [12] showed that the levels of blood urea nitrogen, platelets, hemoglobin and triglyceride were higher in the DMF exposure group (elderly residents living near synthetic leather factories) than in the control group (permanent residents living far away from leather factories). Mice models showed cardiac toxicity of DMF characterised by increased levels of lactate dehydrogenase and creatine kinase-MB partly owing to lipid peroxidation [13]. Zhang et al. [14] found that an alteration of gut microbial community after DMF exposure may cause encephalopathy. The toxic effect on the liver is particularly severe and may cause liver failure, with a high fatality rate [15,16,17]. Although poisoning accidents owing to occupational exposure to DMF have been reported, pathological changes in the liver of these patients have rarely been described. In our centre, a few patients with acute liver failure caused by occupational DMF exposure were successfully treated in recent years and we found that they exhibited specific pathological changes associated with hepatotoxicity. The purpose of the present study was to describe the clinicopathological characteristics of DMF-induced liver toxicity and compare it with a rat liver injury model resulting from intraperitoneal injections of DMF. If representative, such a model can be used to unravel the pathogenesis and test novel treatments.

## 2 Methods

### 2.1 Clinical data collection

Two patients were diagnosed with occupational acute liver failure due to DMF and underwent liver biopsy in 900th Hospital of Joint Logistics Support Force between 2015 and 2018. Details such as general information, occupational exposure history, clinical manifestations, laboratory results, imaging findings, and liver histological examination findings were collected and analysed.

### 2.2 Toxicological model of DMF-induced liver injury

The animal studies were conducted in strict accordance with the recommendations in the Guide for the Care and Use of Laboratory Animals according to the regulation in the People’s Republic of China. The protocol was approved by the Committee on the Ethics of Animal Experiments of 900th Hospital of Joint Logistics Support Force. All animals were sacrificed by peritoneal injection of sodium pentobarbital.

Thirty-six sterile Sprague-Dawley rats aged 6–7 weeks and weighing 200 ± 20 g were purchased from the Zhejiang Provincial Laboratory Animal Centre (Licence Number: SCXK (Zhejiang) 20140001). The animals were kept in the Animal Experimental Centre of the 900th Hospital of Joint Logistics Support Force. After adaptive feeding for 1 week, they were randomly categorised into 3 groups.

#### 2.2.1 DMF group

Twelve rats were injected with DMF (Sinopharm Chemical Reagent, 81007718) intraperitoneally at a dose of 10 mL/kg as a 40% saline solution (final dose 4 g/kg) once a week. At 48 h and 8 weeks, 6 animals in each treatment were euthanised and liver tissue samples were collected.

#### 2.2.2 CCl_4_ group

Twelve rats were intraperitoneally injected with CCl_4_ (Sinopharm Chemical Reagent, cat. no. 10006464) at a dose of 1 mL/kg as a 50% olive oil solution twice a week. At 48 h and 8 weeks, 6 animals in each treatment were euthanised and liver tissue samples were collected.

#### 2.2.3 Control group

Twelve rats were intraperitoneally injected with normal saline at a dose of 10 mL/kg once a week. Euthanasia was conducted after 48 h and 8 weeks.

## 3 Histological processing

The liver tissues including patient and rat samples were fixed in 10% formaldehyde solution overnight and embedded in paraffin before 3 µm thick sections were cut from the tissue blocks. The staining methods and reagents that haematoxylin and eosin (HE) staining, reticular fibre staining, and Masson staining need were conducted according to the standard protocols established by pathology department of 900th Hospital of Joint Logistics Support Force. The staining results were examined independently by two pathologists.


**Ethics approval:** Informed consent for the collection of clinical information was obtained in accordance with the Declaration of Helsinki and this study was approved by the ethics committee of 900th Hospital of Joint Logistics Support Force (2020-044). The written informed consent was obtained from these two patients. Patients and the public were not involved in the design and conduct of our research.

## 4 Results

### 4.1 Clinical cases of hepatic failure exposed to DMF

**Figure 1:**
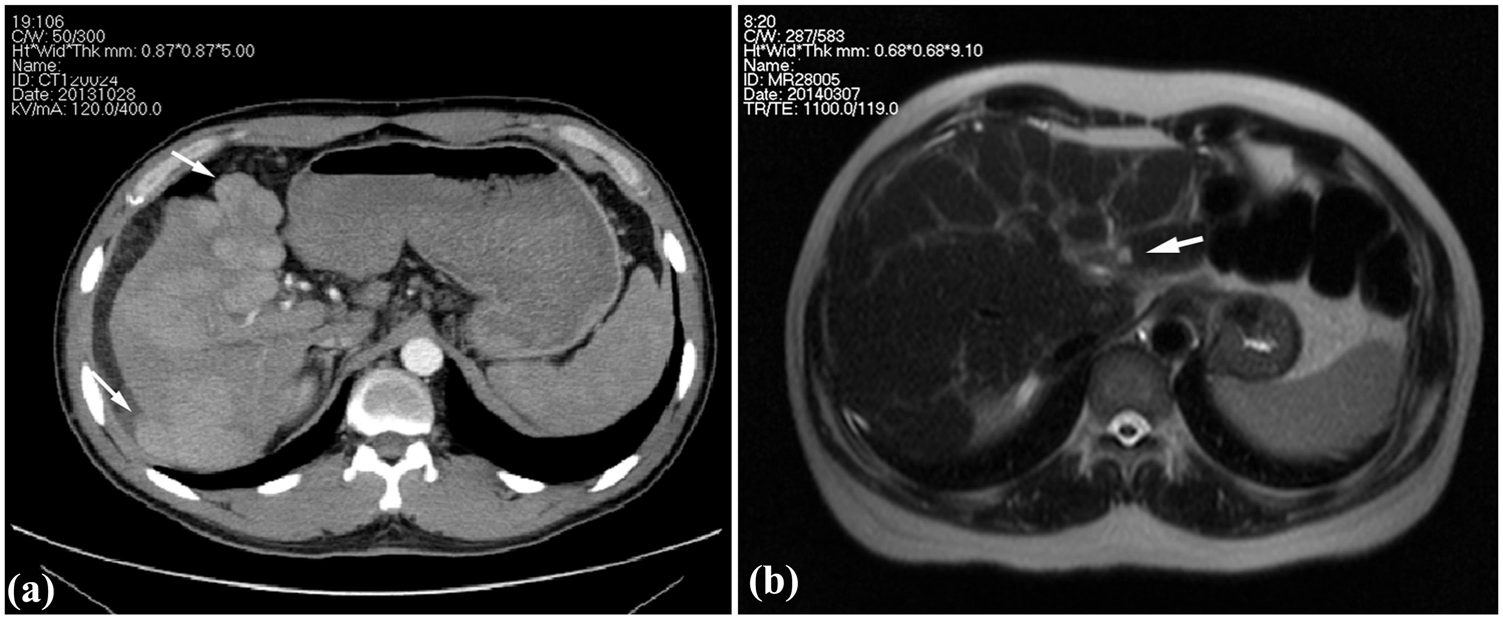
Images of clinical cases. (a) Enhancement CT showing the multiple intrahepatic nodules protruded from the liver surface (arrow) in Case 1 and (b) T2WI of MR showing heterogeneous fibrosis and a nodule in the left inner lobe (arrow) in Case 2.

**Figure A1:**
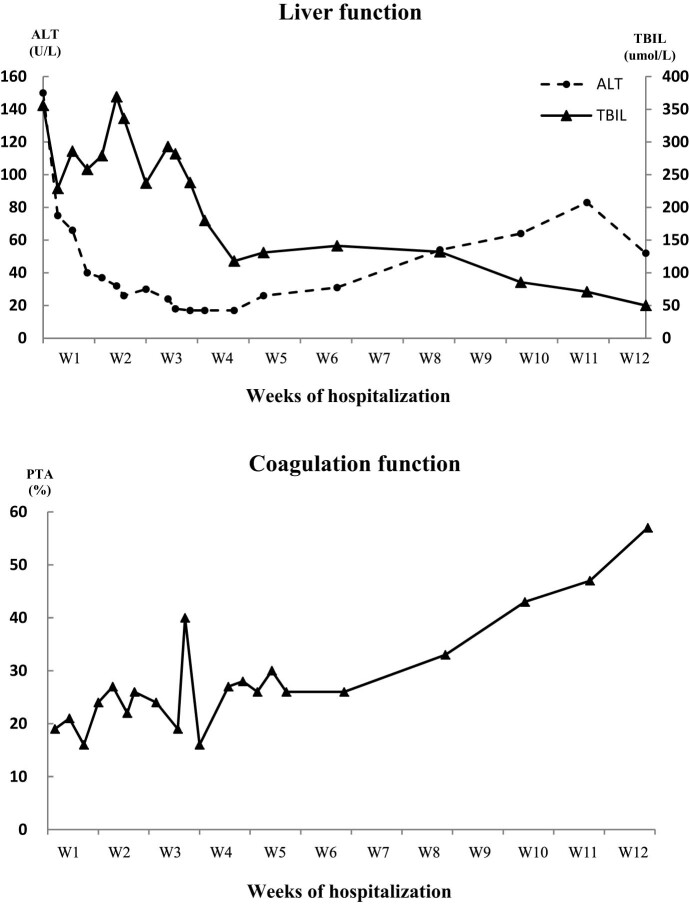
The level of ALT, TBIl and PTA of case 1 during hospitalization.

Case 1: A 36-year-old man was admitted to the hospital with chief complaints of abdominal distention, fatigue, and jaundice that persisted for 2 weeks. The patient had a history of exposure to DMF owing to his job at a leather factory 2.5 months before admission (the first 2 months of drying operation and the next 0.5 month of stirring operation). The patient experienced somnolence and was found to be positive for asterixis. The whole-body skin and sclera had turned severely yellow, with petechiae and ecchymosis noted in both lower limbs. The abdominal shifting dullness was positive. The biochemical analysis revealed: alanine aminotransferase (ALT) 589 U/L, aspartate aminotransferase (AST) 559 U/L, total bilirubin (TBIL) 411 µmol/L, direct bilirubin (DBIL) 273.6 µmol/L, prothrombin activity (PTA) 23%, and fibrinogen 0.83 g/L. Results of serological tests for hepatitis virus, cytomegalovirus, and EB virus were negative. Autoimmune antibodies, ceruloplasmin, and anti-hepatic antigens were not detected. No complications of other organ damage were found. Thus, there were indications of acute severe dimethylformamide poisoning, acute liver failure, and hepatic encephalopathy (phase A2). After admission, the patient received anti-hepatic encephalopathy, infusion of albumin and plasma, prothrombin complex, plasma exchange, and other therapies, and after 2 weeks, he gained consciousness. After 123 days of treatment, liver and coagulation functions returned to normal at discharge (the tendency chart can be seen in Figure A1). On the 78th day of admission, according to the enhancement CT, multiple intrahepatic nodules were significantly enlarged, evenly enhanced in the arterial phase (Figure 1a), and isopycnic with peripheral liver tissues in the portal and delayed phases. Malignant lesions could not be excluded [18]; therefore, a percutaneous liver biopsy was conducted on the 83rd day after obtaining informed consent.

Case 2: A 50-year-old man was admitted to the hospital with chief complaints of abdominal distention, abdominal pain, and poor appetite with yellowing of the skin and sclera that persisted for 1 week. The patient was employed at the local leather factory 5 months before the onset (the first 4 months of drying operation and the next 1 month of coating process). He had a history of exposure to DMF and no alcohol abuse. The shifting dullness was positive. Serological tests for hepatitis virus, cytomegalovirus, and EB virus, autoimmune antibodies and ceruloplasmin were all negative. Biochemical investigations revealed: ALT 321.2 U/L, AST 304.5 U/L, TBIL 231.8 µmol/L, DBIL 127.3 µmol/L, PTA 27%, and fibrinogen 1.06 g/L. The patient was diagnosed with acute severe dimethylformamide poisoning and acute liver failure. Liver protection drugs, infusion of plasma and albumin, and plasma exchange were administered after admission. The symptoms improved, and jaundice faded away, with the PTA gradually recovering. The patient was discharged from the hospital 33 days after admission, returning for re-examination 40 days after discharge. Liver function and coagulation function were normal (the tendency chart can be seen in Supplement Figure 2). Hepatic MR enhancement scan showed reduced liver volume, wavy edge, and an imbalanced proportion of the liver lobe with heterogeneous fibrosis (Figure 1b). Multiple intrahepatic patchy signals, with long T1 signals and slightly long T2 signals, showed enhanced signals after enhanced scan; hence, a percutaneous liver aspiration biopsy was conducted 73 days after the onset.

**Figure 2:**
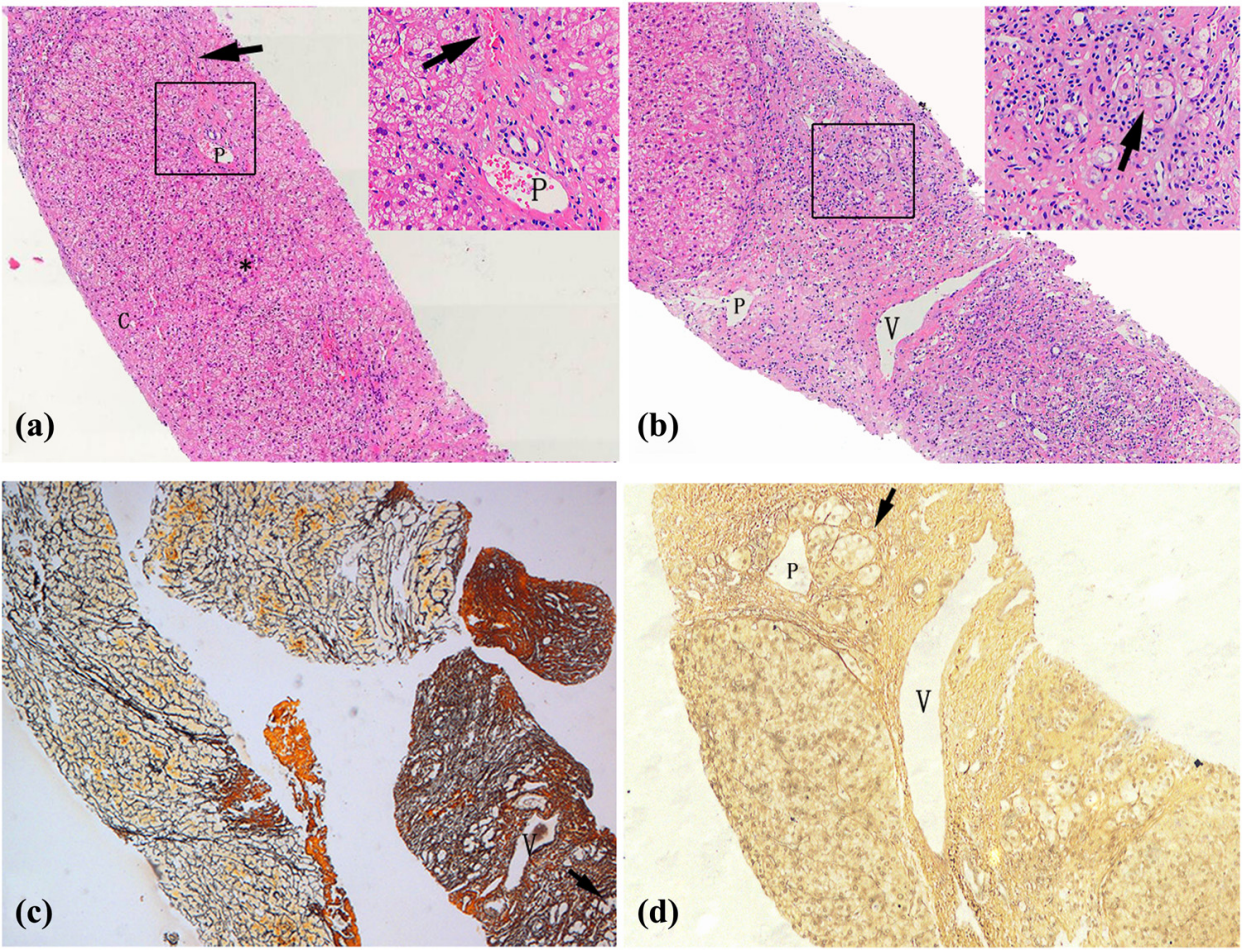
Pathological characteristics of liver tissue in patients. (a) Liver cells with diffuse oedema, balloon-like degeneration, dotted/focal necrosis (*), and local bridging necrosis (arrow) in Case 2 (HE 40× and HE 200×). (b) Hepatic lobules were replaced by proliferative fibrous tissues with hepatocyte regeneration (arrow) in Case 2 (HE 40× and HE 200×). Uneven fibrosis of liver tissues and clusters of regenerative cells (arrow) surrounded by reticulum fibres in Case 1 (c, Reticulum 20×) and Case 2 (d, Reticulum 40×). C: central veins, V: hepatic vein, P: portal vein.

Clinicopathological changes in the livers of both patients were mainly characterised by multiple unevenly distributed lesions of inflammation and fibrosis. Some liver tissues exhibited features of moderate chronic hepatitis (Figure 2a), accompanied by partial balloon-like degeneration, focal necrosis, local bridging necrosis, or different sizes of steatosis on HE staining. In other parts of the liver tissue, the pathological presentation was dominated by fibrosis, in which the hepatic lobules were replaced by significantly proliferative fibrous tissues (Figure 2b). Further, reticulum fibre staining showed local fibrosis and clusters of regenerative cells with clear cytoplasm surrounded by reticular fibres in two cases (Figure 2c and d).

### 4.2 Hepatic injury induced by DMF in animal models

To further observe and verify this specific pathological feature, we successfully created an animal model of DMF-induced liver injury. Meanwhile, as a positive control, CCl_4_ treatment was used to induce the classic model of liver fibrosis.

Compared with the CCl_4_ group, the rats in the DMF group were more depressed, hypoactive, and insensitive to external stimuli. None of the above symptoms were found in the negative control group. Probably because of drug toxicity, one rat each in the DMF group died after 36 h and 7 weeks, respectively, and two rats died in the CCl_4_ group in the seventh week. No rats died in the negative control group.

**Figure 3:**
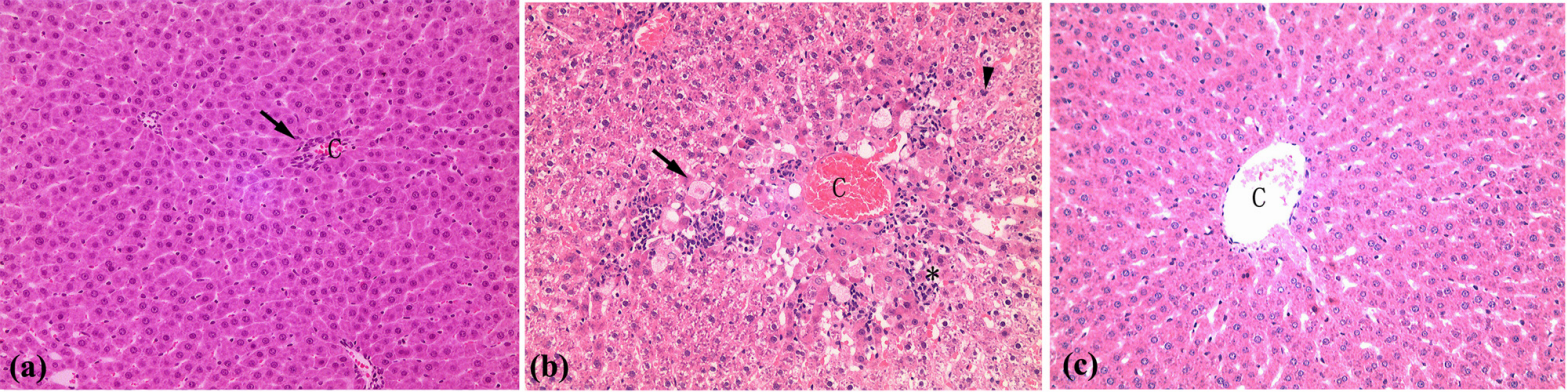
Pathological changes in the liver after administration of for 48 h. (a) The lobules were intact with inflammatory cells infiltrating (arrow) in central veins (DMF group, HE 100×). (b) Balloon-like degeneration and steatosis (arrow), spot-like necrosis (*), and partial apoptosis (triangle) of hepatocytes around the central vein, accompanied by some inflammatory cell infiltration (CCl_4_ group, HE 100×). (c) Normal hepatocytes and hepatic lobular structure (Control group, HE 100×). C: central veins.

In the acute phase, liver inflammation in the DMF group was less severe than that in the CCl_4_ group. At 48 h, 6 rats were euthanised and liver tissues were stained using HE. DMF group exhibited occasional focal-point-like necrosis and slight swelling of central lobular hepatocytes, with partial inflammatory cells infiltrating the portal area and surrounding central veins (Figure 3a). In the CCl_4_ group, diffuse hepatocyte degeneration was observed, including balloon-like degeneration and steatosis, spot-like necrosis, and partial apoptosis of hepatocytes around the central vein in area 3, accompanied by some inflammatory cell infiltration. Furthermore, there were a small number of inflammatory cells infiltrating the portal area (Figure 3b).

**Figure 4:**
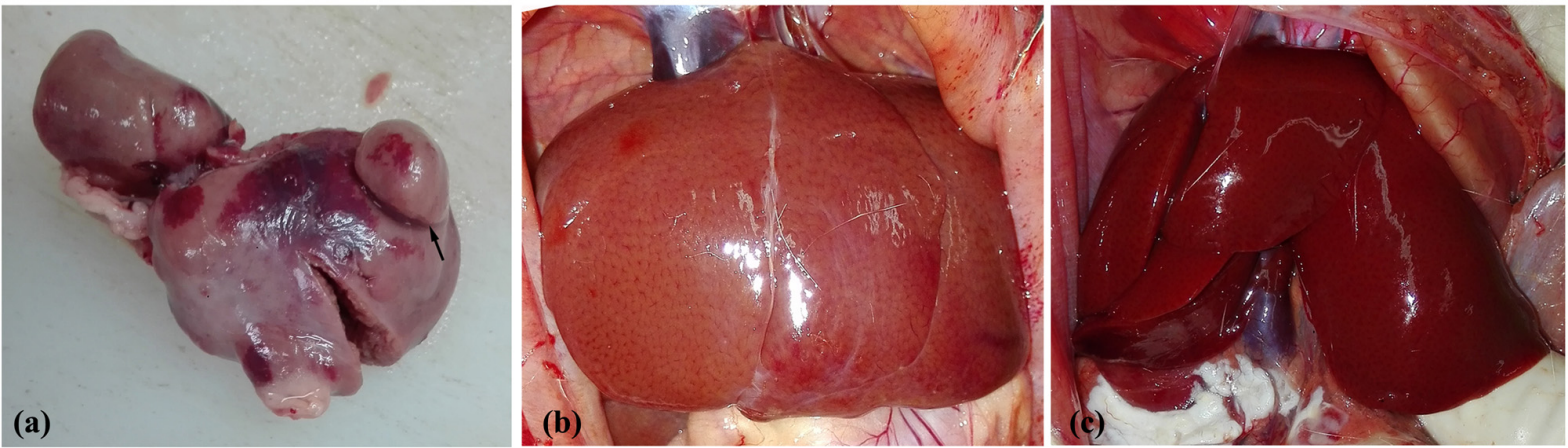
Appearance of the rat liver. (a) Liver tissue protruded from the liver surface (arrow) at week 8 in the DMF group. (b) The liver surface was spotted with a round and blunt edge, and the liver lobe proportion was unbalanced at week 8 in the CCl_4_ group. (c) The livers in the control group were normal, smooth, and soft texture.

By the eighth week, rats were sacrificed to assess chronic liver injury after modelling. The liver surface showed a round and blunt edge, and the liver lobe proportion was unbalanced in both DMF and CCl_4_ groups (Figure 4a and b). The liver capsule in the DMF group was attached to the flatulent intestinal tract, whereas the intraperitoneal adhesions and flatulence in the CCl_4_ group were light. Furthermore, a portion of the liver tissue protruded from the surface in the DMF group, whereas no such changes were observed in the CCL4 group.

**Figure 5:**
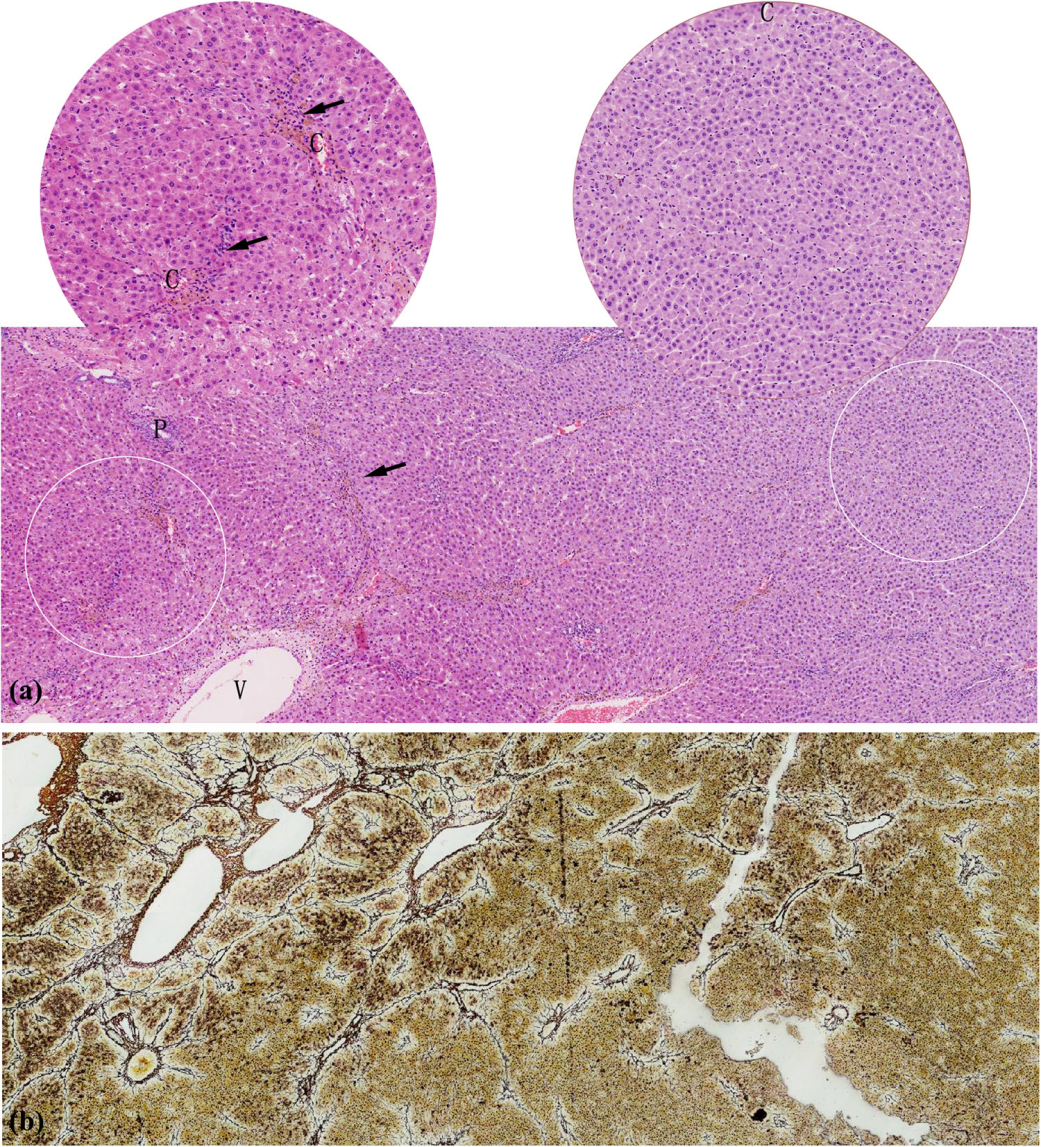
Pathological changes in the liver after administration for 8 weeks in DMF group. (a) Uneven lesions with cell degeneration and narrowed hepatic sinuses in some areas, necrosis, and inflammation (arrow) in other areas (HE 40× and HE 100×). (b) Heterogeneous fibrosis was present in the same pathological section (Reticulum Masson 20×). C: central veins, V: hepatic vein, P: portal vein.

**Figure 6:**
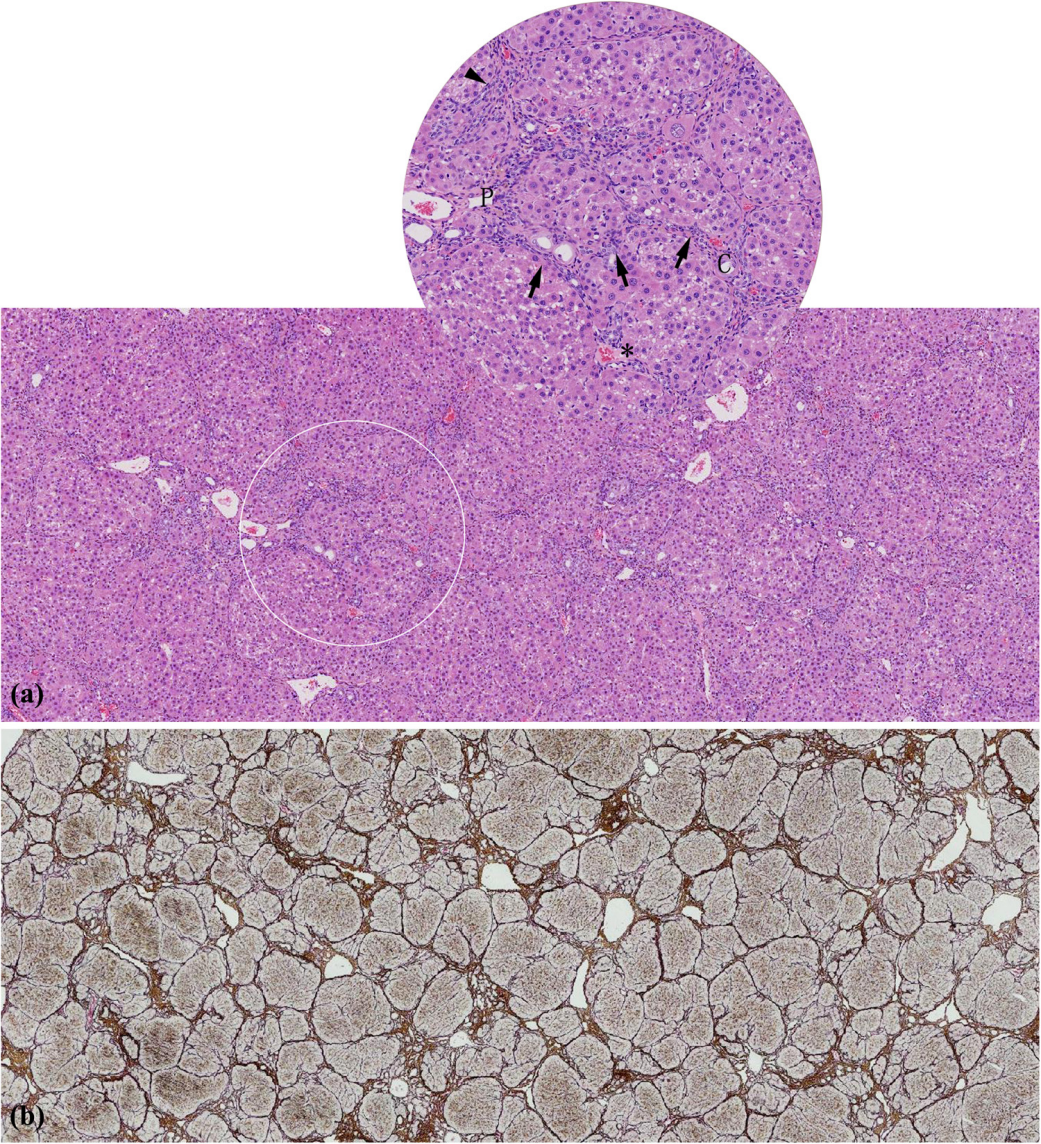
Pathological changes in the liver after administration for 8 weeks in CCl_4_ group. (a) Disordered lobules with extensive degeneration of hepatocytes, patchy coagulation necrosis (*), interlobular fusion necrosis (triangle), and portal area-central vein bridging necrosis (arrow), accompanied by a large amount of inflammatory cell infiltration (HE 40× and HE 100×). (b) Diffuse proliferation of fibrous tissues between hepatic lobules (Reticulum Masson 20×). C: central veins, P: portal vein.

**Figure A3:**
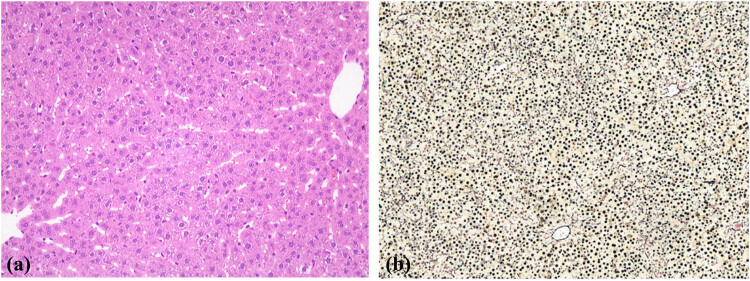
Liver pathology of the control group. The structure of the liver tissue was normal, with no fibrous tissue hyperplasia ((a) HE100×, (b) Reticulum Masson40×).

The liver tissues of the DMF group exhibited varying and heterogeneous extent of degeneration or necrosis and heterogeneous distribution of fibrosis (Figure 5a and b). The HE staining exhibited some structures of hepatic lobules that were intact with swollen and degenerated hepatocytes alone and some structures that were disordered with focal or lytic necrosis of hepatocytes around the central vein, accompanied by inflammatory cell infiltration (Figure 5a). Double staining of reticulum and Masson showed heterogeneous fibrosis: the intact nodules formed by the arcuate connection among the surrounding normal hepatic lobules, the bridging fibres formed in the adjacent portal area and absence of fibrous tissue hyperplasia in the portal area all appeared in the same pathological section (Figure 5b). In contrast, in the CCl_4_ group, liver tissues showed entirely homogeneous and significant inflammation and diffuse homogeneous distribution of micronodular cirrhosis (Figure 6a and b). The hepatic lobules were structurally disordered, with extensive degeneration of hepatocytes, patchy coagulation necrosis, interlobular fusion necrosis, and portal area-central vein bridging necrosis, accompanied by a large amount of inflammatory cell infiltration (Figure 6a). The diffuse proliferation of fibrous tissues between hepatic lobules was observed under reticulation-Masson staining, forming thick fibrous septum connecting portal vein and central vein, as well as segmenting the hepatic cell mass to form small nodular cirrhosis with round, square, or irregular shape being observed (Figure 6b). The structure of the liver tissue in the negative control group was normal, and no inflammation or fibrosis was observed (Figure A3).

## 5 Discussion

DMF is a known hepatotoxic chemical capable of volatilisation at room temperature, and it can be absorbed through the skin and respiratory tract even when wearing gloves and masks [19]. The occurrence of DMF poisoning is closely associated with the concentration of DMF in the working environment and personal protection measures, with a higher incidence of poisoning in high temperature and high humidity conditions [20]. Besides, excessive alcohol consumption, obesity, and chronic liver diseases, such as chronic HBV infection, have also been reported to have synergistic effects with DMF poisoning [21]. Additionally, there is an obvious individual difference in human beings with DMF exposure [22,23].

Although poisoning accidents involving occupational exposure to DMF have frequently been reported, pathological changes in the liver have rarely been described. A previous study reported severe liver injury in a patient exposed to DMF for 2 months. Liver biopsy revealed prominent post-necrotic fibrosis after massive hepatic necrosis, primarily in zone 3, and hepatocyte degeneration in the surrounding tissues, consistent with our study findings [24]. Trevisani et al. [25] also conducted a liver biopsy on a patient with liver failure on the 14th day caused by an overdose of T-61, an animal euthanasia drug with DMF as the solvent, in which DMF contributes to hepatotoxicity. Focal hepatocellular necrosis and early fibrosis were observed within a short time. Considering the cases in this report in combination with other reports, it was suggested that the typical morphological changes in liver fibrosis can occur within a short time of hepatic failure owing to severe DMF poisoning. These morphological changes coincide with regional sub-massive liver necrosis, a special pathological manifestation caused by chemicals [26].

In this study, uneven distribution of multiple inflammation and fibrosis sites was noted in DMF-induced liver injury. Under the microscope, polymorphic inflammation, such as hepatocyte degeneration, necrosis, apoptosis, or regeneration, as well as heterogeneous distribution of fibrosis, were noted simultaneously. This phenomenon was considered a specific liver pathology due to DMF poisoning. As is described above [24,25], patients exposed to large doses of DMF may show similar pathological changes in different time periods. In addition, short exposure and long exposure to DMF may lead to totally different hepatic pathological feature in workers with occupational poisoning [27]. According to Senoh et al. [28], rats were exposed by inhalation to different concentrations of DMF. Massive necrosis associated with centrilobular fibrosis was observed in the liver of 1,600 ppm (v/v) DMF-exposed (6 h/day × 5 days/week) rat 2 weeks later. Only hepatic fatty change and centrilobular necrosis were observed at low concentrations or with the increase in exposure duration. Therefore, we speculate that the specific pathological changes may not be related to the biopsy time point, but is closely related to the concentration and time of exposure to DMF.

In the present study, this specific liver pathology of DMF poisoning is an aid to diagnosis. Liver puncture is not a prerequisite for the diagnosis of DMF poisoning, which is clinically based on a short-term exposure history of a significant amount of DMF, clinical manifestation, laboratory examination of liver damage, and investigation of the work environment after excluding other possible causes of liver damage. For patients with unexplained liver injury, liver biopsy should be conducted as soon as possible if there is no contraindication. Hence, considering special pathological changes in hepatotoxicity in patients, a model of liver injury induced by DMF in rats was established to validate or evaluate liver histopathology.

The DMF concentration required for modelling through inhalation is high, and this cannot truly simulate occupational exposure to DMF. Additionally, subcutaneous administration of DMF is more irritating as it causes skin erosion and ulceration [29]. Therefore, this study was modelled by intraperitoneal injection, which simulates toxin-induced liver fibrosis. It has been found that the median lethal dose of DMF via intraperitoneal injection in rats was 3–7.17 g/kg [30]; meanwhile, previous studies found that a dosage ranging from 0.5 to 1.8 g/kg of DMF could cause liver injury in rats when injected intraperitoneally [30,31,32]. In this study, to induce liver damage for the activation of fibrogenesis without high mortality, we repeated the preliminary experiments by intraperitoneally increasing the dose and finally discovered that rats injected with DMF once a week at a dose of 4 g/kg can develop liver fibrosis at the eighth week. CCl_4_ is currently the most used standard drug for creating liver fibrosis animal models as it can be administered through various routes and has the advantages of simplicity and low cost [33]. Therefore, the administration of CCl_4_ was used as the positive control.

Although DMF and CCl_4_ are both chemical substances, the pathological characteristics of liver damage caused by these are different. In the present study, we found hepatocyte injury and inflammatory response were mild, while hepatocytes were extensively denatured, necrotic, and the inflammatory was prominent in the CCl_4_ group. Fibrosis distribution was not uniform and the fibrous cords were thin in the DMF group. In contrast, CCl_4_-induced hepatic fibrosis was diffuse, with the formation of pseudolobules throughout the field of vision, and the fibre strands were thick.

The reason for the specific pathological changes may be attributed to the toxic effect of DMF [34,35,36]. Most scholars believe that methylisocyanate (MIC), a metabolite of DMF catalysed by CYP2E1 in hepatocytes, plays a potentially vital role in pathogenicity. It undergoes electrophilic activity and can covalently bind with proteins, nucleic acids, and other cellular macromolecules, thereby causing cell damage. Meanwhile, part of MIC can consume glutathione, which could weaken the antioxidant function *in vivo* and cause lipid peroxidation. After intraperitoneal injection of DMF, the drug enters the liver parenchyma through the portal vein. The uneven distribution of the drug and its metabolites in the structural unit of the hepatic acinar may lead to uneven liver injury. Because DMF is less toxic than CCl_4_, liver cells are mainly degenerative without prominent necrosis and inflammatory infiltration in the lobules and portal area is not obvious. Heterogeneous fibrosis was accompanied by uneven inflammation. Collagen fibres formed are thin and mainly distributed in the portal area. During the late stage, there was no fibrosis interval reconstruction of hepatic lobules after massive necrosis of liver cells. However, diffuse distribution of hepatic lobules of fibrous tissue can be observed around the large veins, owing to reduced blood supply, severe ischaemia and hypoxia in this area, which results in post-necrotic cirrhosis.

In conclusion, we first found that uneven distribution and polymorphism of liver inflammation and fibrosis were detected in both humans and animals exposed to DMF. When encountering liver damage of unknown aetiology, the specific pathological finding could be a useful clue for the clinical diagnosis of DMF poisoning. Moreover, to our knowledge, an animal model of DMF-induced liver fibrosis has been established for the first time. However, the mechanism remains unclear. Hence, further studies in clinical and animal experiments are required. Additionally, varying extent and distribution of liver fibrosis including no fibrosis, mild–severe fibrosis and cirrhosis in this model could occur simultaneously in the same individual, which can provide self-contrast advantages in liver fibrosis research on the pathogenetic and progression mechanism to avoid individual differences of animals.

## Figures and Tables

**Figure A2:**
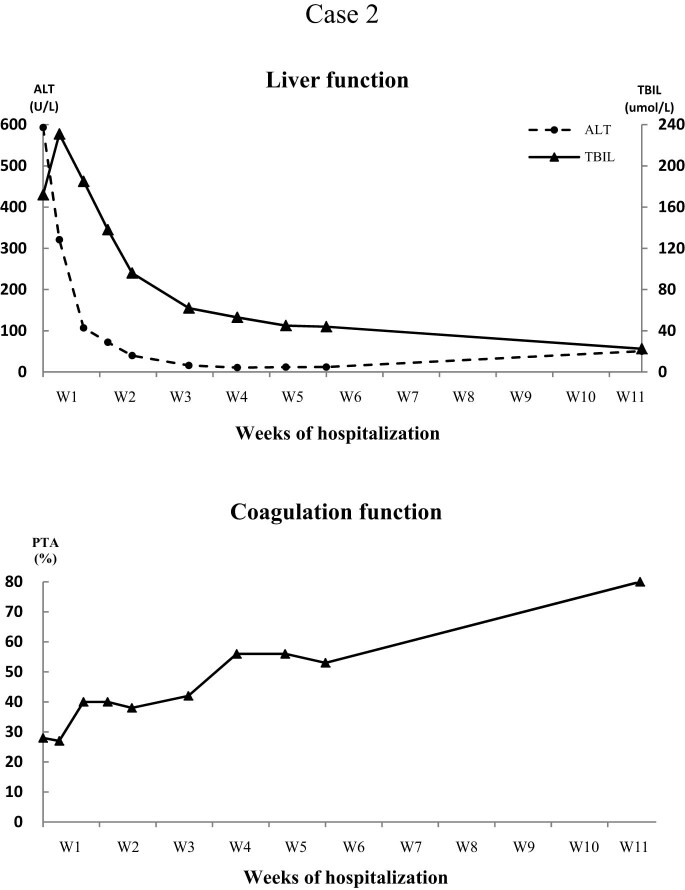
The level of ALT, TBIl and PTA of case 2 during hospitalization.
